# Draft genome sequence of a methicillin-resistant *Staphylococcus aureus* strain isolated from a chronic wound

**DOI:** 10.1128/mra.00431-24

**Published:** 2024-08-23

**Authors:** Kira N. Allison, Rayhane Mejlaoui, Katrina G. DeZeeuw, Jonah E. Marek, Edana Cassol, Joerg Overhage

**Affiliations:** 1 Department of Health Sciences, Carleton University, Ottawa, Ontario, Canada; 2 Department of Complex Continuing Care, Saint Vincent Hospital, Ottawa, Ontario, Canada; University of Maryland School of Medicine, Baltimore, Maryland, USA

**Keywords:** chronic wound, pressure injury, MRSA, Oxford Nanopore sequencing

## Abstract

We report the draft genome sequence and antimicrobial resistance gene profile of a methicillin-resistant *Staphylococcus aureus* (MRSA) clinical isolate recovered from a chronic pressure injury wound infection of an adult female patient. The draft genome sequence included a 2.86-Mb chromosome, which was accompanied by a 27-kb plasmid containing *blaZ*.

## ANNOUNCEMENT

Chronic wounds that do not heal have emerged as a clinical problem and socio-economic burden to the healthcare systems worldwide (1). A barrier to wound healing in chronic wounds is the presence of biofilms, which are characterized by high antimicrobial tolerance and are difficult to treat (2). Gram-positive *Staphylococcus aureus* is among the most frequently isolated pathogens from chronic wounds (1). It has been shown that strain-level variation of *S. aureus* was associated with disease severity and poorer patient outcome (3).

*S. aureus* strain PS00642 was isolated from a culture swab taken from the center of a pressure injury wound from an adult female patient in Ottawa, Canada. The isolate was selected from Oxacillin Resistance Screening Agar (Dalynn Biologicals) supplemented with 2 mg/L oxacillin +5 mg/L polymyxin B after 37°C incubation for 48 hours. Determination of the minimal inhibitory concentration (MIC) by broth dilution assay (4) revealed an MIC for oxacillin of 256 (μg/mL), demonstrating that *S. aureus* strain PS00642 is a methicillin-resistant *S. aureus* (MRSA) strain. For whole genome sequencing, cells were grown at 37°C for 18 hours at 280 rpm in LB media. DNA was extracted using the PureLink Microbiome DNA Purification Kit (Invitrogen) following the protocol optimized for urine and microbial culture extraction. Samples were prepared for Oxford Nanopore Technologies (ONT) using the Rapid Sequencing Kit V14 (SQK-RAD114, ONT) and were sequenced with a MinION (Mk1B) and a Flongle R10.4.1 (FLO-FLG114) for 24 hours. MiniKNOW.v.23.04.3 assessed the read quality (q score >9), and basecaller dorado.v.7.3.9 detected and removed adapter and/or primer sequences. Guppy.v.6.5.7 in high-accuracy mode generated Fastq files. *De novo* genome assembly and overlap detection/trimming of files were performed with flye.v.2.9.3-b1797 (5), variant calling and polishing with medaka.v.1.11.3 (https://github.com/nanoporetech/medaka), and genome annotation with prokka.v.1.14.5 (6). VirulenceFinder.v.2.0 (7) and ResFinder.v.4.1 (8, 9) were used to predict virulence factor genes (VFG) and antimicrobial resistance (AMR) genes. EPI2ME AMR workflow (v2023.04.26-1808834) was used for taxonomic identification and to corroborate AMR results, with input reads aligned against reference sequences in the Comprehensive Antimicrobial Resistance Database (CARD). Default parameters and protocols were utilized unless stated otherwise. Ethical approval for the study was granted by the Carleton University Ethics Committee (118036).

In summary, 51,180 raw sequencing reads with a mean read length of 5,453 kb were used to construct the *S. aureus* chromosome (2,864,045 bp) with a genome coverage of 88× and an estimated N50 of 10.67 Kb. The G+C content of 32.8% is similar to values observed in other studies (10, 11). The NCBI Prokaryotic Genome Annotation Pipeline (v6.7) annotated a total of 2,899 genes. Using the ResFinder and EPI2ME AMR detection, six clinically relevant AMR genes were identified indicating resistance to four antibiotic classes: aminoglycosides (aadD_AF181950), quinolones, macrolides [erm(A)_X03216], and β-lactams (mecA_BX571856). The VirulenceFinder predicted 23 VFGs, 3 exoenzyme, 13 toxin, and 2 hostimm genes. The accompanying plasmid (27,224.5 bp) with a G+C content of 27.2% contained β-lactam resistance gene blaZ_JDXN01000019 and upstream regulators blaR1 and blaI.

## Data Availability

All sample reads can be found under BioProject PRJNA1083987. Sequencing reads of *S. aureus* PS00642 can be found at SRA SRR28440632, BioSample SAMN40603899, and genome submission CP151814 - CP151815.
